# A convenient Simple Method for Synthesis of Meta-iodobenzylguanidine (MIBG) 

**Published:** 2013

**Authors:** Zahra Sheikholislam, Zohreh Soleimani, Abolghasem Moghimi, Soraya Shahhosseini

**Affiliations:** a *Pharmaceutical Chemistry Department, School of Pharmacy, Shahid Beheshti University of Medical Sciences, Tehran, Iran, P O Box: 14155-6153. *; b *Department of Chemistry, University of Imam Hossein. *

**Keywords:** Meta-iodobenzylguanidine, Neuroendocrine tumors, Sympathetic neurons, Radioiodinated MIBG

## Abstract

Radioiodinated meta-iodobenzylguanidine (MIBG) is one of the important radiopharmaceuticals in Nuclear Medicine. [^123/131^I] MIBG is used for imaging of Adrenal medulla, studying heart sympathetic nerves, treatment of pheochromacytoma and neuroblastoma. For clinical application, radioiodinated MIBG is prepared through isotopic exchange method, which includes replacement of radioactive iodine in a nucleophilic substitution reaction with cold iodine (^127^I). The unlabelled MIBG hemisulfate is synthesized by the procedure described by Wieland *et al. *(1980). The availability of a more practical and cost-effective procedure for MIBG preparation encouraged us to study the MIBG synthesis methods. In this study the preparation of MIBG through different methods were evaluated and a new method, which is one step, simple and cost-effective is introduced. The method has ability to be scaled up for production of unlabelled MIBG.

## Introduction

Meta-iodobenzylguanidine (MIBG) a structural and functional analogue of norepinephrine (NE), is transported, stored, and released in the adrenergic neuron by the same mechanisms as NE which includes neuronal (uptake-1) and extra neuronal (uptake-2, myocyte) specific membrane transport process and passive diffusion in and from both cellular compartments. MIBG unlike NE is a stable and non-metabolizable compound in vivo, resistant to monoamine oxidase (MAO) and catechol-o-methyl transferase (COMT) enzyme systems. Studies have shown that the norepinephrine transporter (NET) is highly over expressed in neuroendocrine tumors such as neuroblastoma, pheochromocytoma, paraganglioma, and carcinoid. Since MIBG is a substrate for NET, therefore, MIBG and its radioiodinated forms accumulate in neuroendocrine tumors. In clinic, radioiodinated MIBG is used for treatment and diagnosis of neuroendocrine tumors. Accumulation of radioiodinated MIBG in adrenergic neurons clinically used for non-invasive assessment of sympathetic neuronal functions (1-9). 

Although MIBG has been radiolabelled with radioisotopes such as ^211^At, ^18^F, ^76^Br none of these radiopharmaceuticals are in clinic yet (10-13). The most important radioisotopes for radiolabeling MIBG are still iodine radioisotopes (^123/131^I). Radioiodinated MIBG is an important diagnostic and therapeutic radiopharmaceutical in clinic. For clinical application, radioiodinated MIBG is prepared through isotopic exchange method (14-16). In this method, radioactive iodine (^123/131^I) in a nucleophilic substitution reaction replaces cold iodine (^127^I) in MIBG molecule. The unlabelled MIBG hemisulfate is synthesized by the procedure described by Wieland *et al. *(1980). The availability of a more practical procedure for MIBG preparation encouraged us to study the MIBG synthesis methods. In this study, MIBG was synthesized through 4 different methods. In each method, the product was characterized by melting point and spectroscopic methods (IR, ^1^H-NMR, ^13^C-NMR, Mass). The purity was determined using Reverse phase HPLC and TLC. The spectra data for in-house MIBG was in agreement with structure of MIBG. The purity of MIBG hemisulfate synthesized using meta-iodobenzylamine and S-ethylisothiouronium (method four) was found to be comparable with authentic reference MIBG (Sigma). The synthesis compare to Wieland procedure, which is used for MIBG synthesis, has fewer steps. It is simple and because of production of ethanethiol as a by-product is cost-effective. The new method has the ability to be replaced by traditional methods for preparation of unlabelled MIBG. 

## Experimental

All chemicals were purchased from Merck except meta-iodobenzyamine hydrochloride (MIBA HCl) and reference MIBG which were purchased from Sigma-Aldrich. 

Four different methods were used for synthesizing MIBG. 

Method 1: MIBG hemisulfate was synthesized according to the procedure described by Wieland *et al. *(1980). Briefly, a mixture of MIBA HCl (1 mmole) and cyanamide (1.5 mmole) was refluxed 4 hrs at 100C. The resulting oily product was dissolved in 0.5 mL water and 0.5 mL KHCO_3_ (100 mg/ 0.5 mL) was added slowly with stirring. The precipitated MIBG bicarbonate was filtered by vacuum, washed by cold water, and dried in vacuum oven (M.P 121-124°C, yield: 60%). To a mixture of MIBG bicarbonate (0.6 mmole) in 2 mL water, 0.3 mL 2 N H_2_SO_4_ was added slowly. The MIBG hemisulfate was dissolved in water by heat and crystallized upon cooling at room temperature. The crystals was filtered by vacuum, washed by cold water, and dried in vacuum oven. Re-crystallization was done in a mixture of water-ethanol (50:50) (2). M.P 166-167.5°C, IR (KBr, cm^-1^): 3302 (NH), 3138 (NH), 1656 and 1537 (C=N), 1057 (S=O), 785 and 686 (1, 3-disubstituted benzene). 1H-NMR (D2O/500 MHz): *δ *7.1 (t, ^1^H, phenyl H5), 7.3 (d, ^1^H, phenyl H6), 7.5 (m, 2H, phenyl H2, H4), 4.3 (s, 1H, CH2 Benzyl) ppm. ^13^C-NMR (D_2_O/125 MHz): *δ *40 (CH_2_-Benzyl), 95 (C_3_-phenyl), 130 (C6-phenyl), 135 (C5–phenyl), 137 (C4- phenyl), 140 (C2- phenyl), 142 (C1- phenyl), 158 (C-Imine) ppm. MS (ESI): 276 (M+H^+^). 

Method 2: To a solution of 3.3 g sodium (Na) in 20 mL super dry ethanol, 2 mmole guanidine HCl was added, and refluxed for 15 min. After filtration of sodium chloride, 2 mmole meta-iodobenzylbromide in anhydrous ethanol was added to filtrate and refluxed for 36 hrs. Solvent was removed by rotary evaporation. MIBG was crystallized in methanol (17-18). Yield was 43%. IR (KBr, cm^-1^): 3367 and 3162 (NH), 1636 and 1560 (C=N), 801 and 737 (1,3-disubstituted benzene). 1H-NMR (CDCl_3_/500 MHz): δ 7 (t, 1H, phenyl H5), 7.1 (d, 1H, phenyl H6), 7.3 (m, 2H, phenyl H2, H4), 4 (s, 1H, CH2Benzyl) ppm. 13C-NMR (CDCl_3_/125 MHz): δ 40 (CH_2_- Benzyl), 93 (C3- phenyl), 130 (C6- phenyl), 135 (C5- phenyl), 135 (C4- phenyl), 139 (C2- phenyl), 142 (C1- phenyl), 158 (C-Imine) ppm. MS (ESI): 276 (M+H^+^). 

Method 3: A mixture of 0.8 mmole cyanoguanidine with 2 mmole MIBA HCl was heated for 6 h at 200°C while purging with nitrogen gas. MIBG was crystallized upon cooling (19). Yield was 60.5%. IR (KBr, cm^-1^): 3298 and 3011(NH), 1685 and 1628 (C=N), 841 and 697 (1,3-disubstituted benzene). 1H-NMR (CDCl_3_/500 MHz): δ 7 (t, 1H, phenyl H5), 7.3 (d, 1H, phenyl H6), 7.5 (m, 2H, phenyl H2, H4), 3.9 (s, ^1^H, CH_2_ Benzyl) ppm. 13C-NMR (CDCl_3_/125 MHz): *δ *40(CH_2_-Benzyl), 95 (C3-phenyl), 125 (C6-phenyl), 130 (C5-phenyl), 135 (C4- phenyl), 137 (C2- phenyl), 139 (C1- phenyl), 160 (C-Imine) ppm. MS (ESI): 276 (M+H^+^). 

Method 4: To a mixture of 0.7 mmole MIBA HCl in 15 mL water, 0.3 mmole S-ethylisothiouronium sulfate [NH_2_C(NH) S-Et+]_2_SO_4_^2^- was added gradually. The mixture was stirred 5 hrs at room temperature. Water was removed by rotary evaporation and the precipitate was crystallized in acetone. Re-crystallization was done on acetone. Ethanethiol a valuable by-product of reaction was collected during the reaction. Yield was 63%. IR (KBr, cm^-1^): 3339 and 3274 (NH), 1643 and 1662 (C=N), 1092 (S=O), 887 and 701 (1,3-disubstituted benzene). 13C-NMR (D_2_O/125 MHz): δ 40 (CH_2_-Benzyl), 95 (C3- phenyl), 125 (C6- phenyl), 127 (C5- phenyl), 130 (C4- phenyl), 133 (C2- phenyl), 135 (C1- phenyl), 165 (C-Imine) ppm. MS (ESI): 276 (M+H^+^). 

Synthesis of S-ethylisothiouronium sulfate [NH_2_C(NH)S-Et]^2+^SO_4_^2-^ was done based on method of Osklo-Salmani A, 2003. Briefly, a reaction mixture of 0.15 mole diethylsulfate and 0.2 mole thiourea was stirred at 100°C for 2.5 hrs and then at 140°C for an additional 1.5 hrs. Upon cooling, S-ethylisothiouronium sulfate was obtained with melting point 219-222°C and 93% yield (20). IR (KBr, cm^-1^): 3385, 3140, 2820, 2125, 1620, 1405, 1380, 1330, 1230, 1080, 1000, 920, 870. 13C-NMR (DMSO-d6, D_2_O/125 MHz): δ 172.1 (C=N), 25.9 (SCH_2_), 13.9 (CH_3_) ppm. 

Quality control of synthesized MIBG was done through RP-HPLC and TLC based on United States Pharmacopeia USP (21, 2008). HPLC (Merck-Hitachi, L7100, L7420): C18 Chro.Monolithic 100-4.6 mm, mobile phase: 3.04 g triethylamine per 1 liter of Water: Acetonitrile (90:10), pH 4. Flow rate: 1.5 mL/min, 229 nm. TLC: Silica gel, HCCl_3_: CH_3_OH (95:5). 

## Results and Discussion

Radioiodinated MIBG is one of the important radiopharmaceuticals in Nuclear Medicine. [^123/131^I]MIBG is used for imaging of Adrenal medulla, studying heart sympathetic nerves, treatment of pheochromacytoma and neuroblastoma. For clinical application, radioiodinated MIBG is prepared by exchange of radioactive iodine with non-radioactive one using isotope exchange method. 

The method used for synthesis of unlabelled MIBG is based on Wieland’s procedure (2). In this method, the reaction of MIBA HCl and cyanamide in the presence of KHCO_3_, results in MIBG bicarbonate formation. By adding H_2_SO_4_ to MIBG bicarbonate, MIBG hemisulfate is crystallized. A few times re-crystallization in a mixture of water:ethanol (50:50) is used for purification of MIBG hemisulfate. 

**Figure 1:**
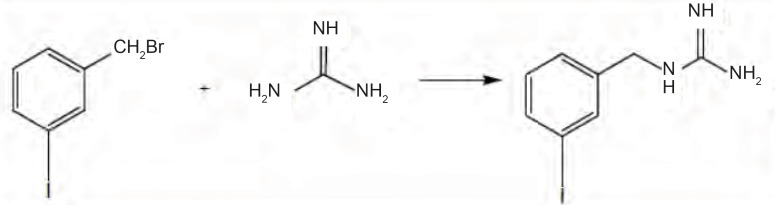
Scheme for the synthesis of MIBG from meta-iodobenzylbromide with guanidine

The reaction of alkyl halide with appropriate amine was used for preparation of MIBG in second method. Guanidine HCl in a solution of Na in ethanol makes a good base to react with m-iodobezylbromide to produce MIBG (Figure 1). Resulting MIBG was crystallized on methanol. Yield was 43%. This method does not have any advantages to Wieland procedure. 

**Figure 2:**
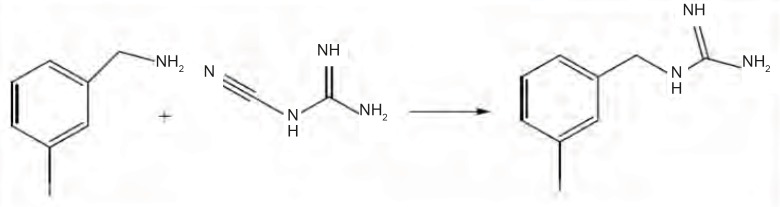
Scheme for the synthesis of MIBG from meta-iodobenzylamine with cyanoguanidine

The reaction of cyanoguanidine with ammonium salts, which results in guanidinium salts, was used in third method for MIBG preparation. Mixture of Cyanoguanidine and MIBA HCl was heated up 200°C for 6 hrs (Figure 2). Crystals of MIBG was formed upon cooling. The yield was 60.5%. Since no solvent is used in this reaction, there is no step to eliminate solvent which is advantageous of this method. 

The reaction of MIBA HCl with salt of S-ethylisothiouronium sulfate produced MIBG sulfate in one step. Yield was 63%. Ethanethiol, the by-product, was collected which is used for synthesis of other chemicals. In this method, MIBG hemisulfate is formed directly without adding H_2_SO_4_. 

Comparing the methods, method 2 and 3 are similar and do not have any specific advantages to Wieland procedure which is routinely used to synthesis MIBG sulfate. Method four is a new method which is reported for first time. In this method, MIBG hemisulfate is prepared through one step. The by-product of reaction is easily collected and is used in industry for synthesizing other chemicals. Considering yield, cost, steps, and simplicity the new method looks promising to be replaced instead of Wieland procedure.

In summary, a convenient and simple method for the synthesis of MIBG was developed. The method has ability to be scaled up for production of unlabelled MIBG. 
